# The effect of high-intensity interval training on cardiorespiratory fitness for non-ambulatory stroke survivors: a randomized controlled trial

**DOI:** 10.3389/fspor.2026.1877466

**Published:** 2026-07-31

**Authors:** Jia Hu, Qingqing Yang, Jing Zhang, Jiao Luo, Wenshi Chen, Fubing Zha, Meiling Huang, Yulong Wang, Mingchao Zhou

**Affiliations:** 1 Department of Rehabilitation, The Second People's Hospital of Shenzhen, The First Affiliated Hospital of Shenzhen University, Guangdong, Shenzhen, China; 2 School of Rehabilitation Medicine, Shandong University of Traditional Chinese Medicine, Shandong, Jinan, China; 3 Department of Rehabilitation, Shenzhen Dapeng New District Nan'ao People's Hospital, Guangdong, Shenzhen, China

**Keywords:** cardiorespiratory fitness, high-intensity interval training (HIIT), non-ambulation, peak oxygen uptake, stroke

## Abstract

**Purpose:**

To compare the effects of high-intensity interval training (HIIT) in improving cardiorespiratory fitness (CRF) with low-intensity continuous training (LICT) in non-ambulatory stroke patients.

**Methods:**

40 non-ambulatory patients (33 men) with ischemic or hemorrhagic stroke (mean age 53 years) were randomly allocated to the intervention (*n* = 20) or control group (*n* = 20). Participants in the intervention group underwent HIIT (1 min high-intensity alternated with 1 min low-intensity), and those in the control group underwent LICT. The training protocol was repeated five times per week for four consecutive weeks (20 training sessions). The patients were assessed before and after the intervention. The primary outcome measure was peak oxygen uptake (VO_2peak_). The secondary outcomes were VO_2AT_, modified Rankin Scale(mRS), Barthel index (BI), and Fugl-Meyer assessment lower limb (FMA-LL).

**Results:**

No differences were found between the two groups at baseline for clinical messages or functional levels (*P* > 0.05). BI, and FMA-LL all increased following training in both the HIIT and LICT groups. There was no statistically significant difference in pre-to-post changes in VO_2peak_ across the HIIT and LICT groups (*P* = 0.665). However, Subgroup analysis revealed that among patients with admission BI ≥60, the HIIT group exhibited significantly higher post-intervention VO_2peak_ than the LICT group (16.35 ± 3.53 vs. 12.74 ± 2.60 mL·kg^−1^·min^−1^, *P* = 0.020). Additionally, post-intervention VO_2peak_ in the HIIT group was significantly higher in the normal-weight subgroup (BMI ≤24) than in the overweight subgroup (16.90 ± 3.11 vs. 13.51 ± 2.02 mL·kg^−1^·min^−1^, *P* = 0.016).

**Conclusions:**

Overall, a 4-week short-interval HIIT program was not superior to LICT in improving cardiorespiratory fitness among non-ambulatory stroke patients. Exploratory subgroup analyses suggested that HIIT may confer greater benefit in patients with higher admission activity levels (Barthel Index ≥60) and normal BMI (≤24 kg/m^2^). Future trials with longer durations and larger sample sizes are needed to confirm its precise efficacy and to develop more effective aerobic exercise protocols for non-ambulatory patients.

**Clinical Trial Registration:**

Chinese Clinical Trial Registry, ChiCTR2000035352.

## Introduction

Stroke is a major global public health challenge, ranking as the second leading cause of death and third leading cause of disability worldwide (1). Neurological dysfunction is the most common impairment among stroke survivors, however, post-stroke cardiopulmonary dysfunction has received increasing attention in recent years (2). Existing evidence indicates that the peak oxygen uptake (VO_2peak_) of stroke patients is generally low, ranging from only 8–22 mL·kg^−1^·min^−1^, which corresponds to 26% to 87% of that in age-matched healthy individuals (3). The underlying mechanisms behind this phenomenon may involve two aspects: first, neurogenic cardiopulmonary complications directly caused by the brain injury itself, second, a sedentary state or prolonged bed rest resulting from persistent motor dysfunction, which further accelerates the progressive decline of CRF (4, 5). Research demonstrates that just 10 days of bed rest and immobilization can reduce CRF by up to 12% (6). Given that reduced CRF is closely associated with higher complication rates, increased risk of early mortality, and poor functional prognosis, exploring effective rehabilitation strategies to improve cardiorespiratory fitness in stroke patients carries significant clinical importance (7).

Aerobic exercise is one of the core interventions for improving cardiometabolic risk factors (8). Traditional continuous aerobic training has played an important role in enhancing cardiorespiratory fitness and improving functional mobility, and has been widely applied in clinical practice. However, stroke patients often have difficulty sustaining high-intensity exercise loads for prolonged periods due to physical disability, fatigue, and impaired balance, which limits the application of traditional training modalities in this population (9, 10). Against this background, high-intensity interval training (HIIT) has attracted increasing attention (11). Characterized by alternating periods of high-intensity exercise and low-intensity recovery, HIIT aims to maximize total exercise volume and cardiovascular stimulation at a lower time cost (12). Its underlying mechanisms, on one hand, are similar to traditional continuous aerobic exercise, inducing central and peripheral cardiovascular adaptations by promoting skeletal muscle perfusion and enhancing myocardial contractility, on the other hand, HIIT may play a more prominent role in promoting mitochondrial biogenesis and fat oxidation metabolism (13–16). Compared to traditional aerobic training, HIIT also has advantages such as better compliance, flexible protocol design, and ease of individualized adjustment (12).

Existing evidence suggests that even patients with severely impaired physical function can benefit from HIIT using equipment such as stationary cycle ergometers, body-weight-supported treadmill training systems, and recumbent whole-body cross-trainers (17, 18). For instance, total-body-linked HIIT has shown high compliance and effectively improved walking distance in frail elderly individuals (19). Early bicycle training used in intensive care patients can also improve overall cardiopulmonary function and facilitate weaning from mechanical ventilation (20). However, current HIIT research in the stroke field focuses predominantly on ambulatory patients and primarily concerns improvements in gait parameters, and its core effects on cardiorespiratory fitness remain controversial across different studies (21, 22). For example, in a short-interval HIIT study conducted by Boyne et al., the difference in VO_2peak_ improvement between the intervention and control groups did not reach statistical significance (23), suggesting that the impact of HIIT on cardiorespiratory fitness in the stroke population still warrants further verification.

More crucially, no study to date has specifically evaluated the effects of HIIT in non-ambulatory stroke patients. Due to severely impaired walking function, this population exhibits a more pronounced decline in cardiorespiratory fitness, while facing greater physical barriers when participating in conventional moderate-to-high-intensity aerobic training, making them a high-risk group that urgently requires clinical attention yet has long been overlooked. Based on this, the present study intends to conduct a randomized controlled trial to compare the effects of HIIT vs. LICT on the cardiorespiratory fitness of non-ambulatory stroke patients, aiming to provide evidence-based support for formulating cardiopulmonary rehabilitation protocols for this severely function-impaired population.

## Materials and methods

This prospective randomized controlled trial (RCT) compared a short-interval HIIT protocol (intervention) with a LICT protocol (control) among non-ambulatory stroke patients in the rehabilitation center of Shenzhen Second People's Hospital, China. The study protocol was approved by the Ethics Committee of the First Affiliated Hospital of Shenzhen University (reference no.20203357019-XZ2021) and registered in the Chinese Clinical Trials Registry Platform (identifier: ChiCTR2000035352). This study used the CONSORT reporting guidelines (24).

Based on the sample size calculation method for a pilot RCT study, a minimum sample size of 18 participants in each group was needed to achieve 90% power and detect an effect size (Cohen's f 2) of 1.14 attributable to two independent variables using a t-test (RCT analysis) with a significance level (alpha) of 0.05. With a 20% shedding rate, 44 subjects were needed for this study.

### Participants

**Figure 1:**
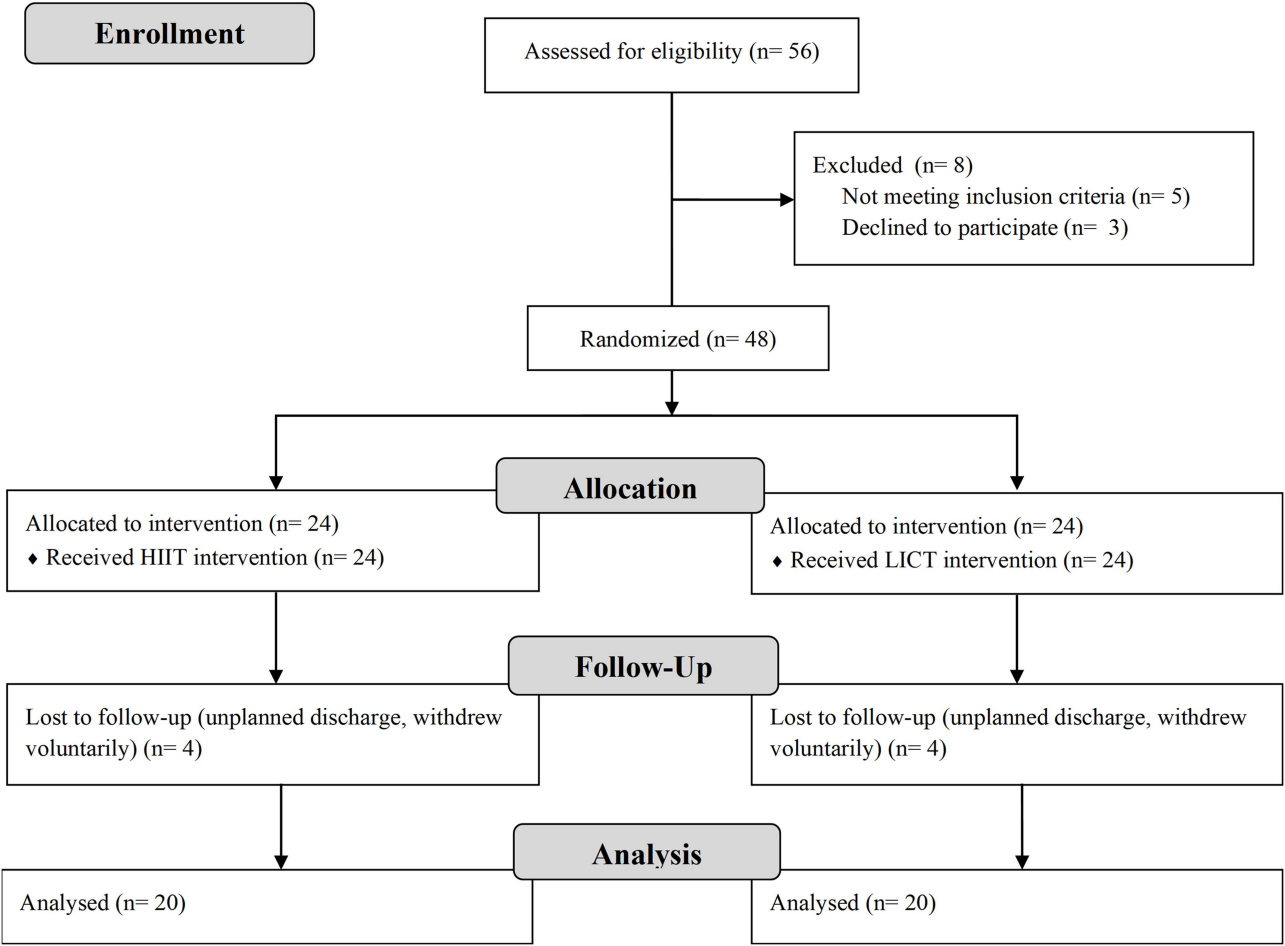
A flowchart of the participants.

Participants were recruited between November 2021 and July 2022. The recruitment advertisement was published in the flyouts of the rehabilitation center. The inclusion criteria were as follows: (1) age between 18 and 75 years; (2) clinically diagnosed with ischemic or hemorrhagic stroke; (3) duration since stroke onset greater than 15 days; (4) medically stable and with no significant pain limitations; (5) modified Rankin grade ranging from 3 to 5; (6) functional ambulation category ≤2; (7) hip extension and knee extension can be actively performed by at least one lower extremity; (8) matching degrees S5Q > 3 points, and can comprehend and actively engage in the examination and training program. The exclusion criteria were as follows: (1) cardiac contraindications for exercise testing according to the American College of Sports Medicine(ACSM) (25); (2) concurrent neurological disease (e.g., multiple sclerosis, Parkinson's disease, etc.); (3) concurrent pulmonary disease (e.g., chronic obstructive pulmonary disease, etc.); (4) other neurological or orthopedic conditions that may cause motor deficits (e.g., fracture, degenerative joint changes, or clinical instability of the hip or knee joint); (5) high muscular tension, Ashworth grade 2 to 4; and (6) uncontrolled hypertension, arrhythmia, or an unstable cardiovascular status as advised by the attending physician. A flowchart of the participants is shown in Figure 1.

### Randomization and blinding

Patients who met the inclusion criteria were informed of the study's purpose and procedure, and written informed consent was obtained before participation. Before recruiting patients, the researchers created a simple computer-generated random sequence using Microsoft Excel. Random sequences were stored in sealed, opaque, consecutively numbered envelopes by an external person uninvolved in the treatment. The researchers opened the envelope in the presence of participants on the first day of treatment, and then the participants were informed of the randomization result. Demographic and baseline characteristics of each participant were collected at the time of enrollment.

### Interventions

All patients in the study underwent routine rehabilitation therapy, including limb functional rehabilitation training, joint mobility training, and balance training.

In addition to standard care, the two groups underwent cardiopulmonary endurance training at different intensities. All training was performed on the same model of recumbent four-limb linkage cross-trainer (Humaneotec Company, Guangzhou) to ensure consistency and comparability of the exercise modality. Each training session lasted a total of 30 min (including a 5-minute warm-up and a 5-minute cool-down) for both groups. Training was conducted once daily, five days per week, for four consecutive weeks, totaling 20 sessions.

Before training, the therapist explained the training methods and precautions to the patients in detail. During training, patients wore a telemetry monitor (C100A, Shenzhen Koman) for dynamic heart rate monitoring and were continuously supervised by a systematically trained therapist familiar with the study protocol, who was responsible for monitoring heart rate changes and adjusting training intensity in a timely manner. Heart rate and blood pressure were measured and recorded before and after each training session. If adverse events such as chest tightness, shortness of breath, dizziness, or syncope occurred during training, the session was immediately stopped, emergency procedures were initiated if necessary, and the event was recorded in detail in the case report form.

#### Intervention group: high-intensity interval resistance training (HIIT)

Exercise pattern: 5-minute warm-up, followed by 20 min of high-intensity interval training, and a 5-minute cool-down. The 20-minute main training phase used 10 cycles of alternating 1-minute high-intensity bursts and 1-minute active recovery periods (1 × 10 structure). During the high-intensity phases, patients were required to achieve a heart rate of 85%–95% of peak heart rate (HR_peak_), and during the active recovery phases, heart rate was controlled at 50%–70% HR_peak_. The therapist continuously monitored heart rate, and if the patient did not reach the target zone, instructed the patient to increase pedaling speed to maintain the workload intensity.

#### Control group: Low-intensity continuous resistance training (LICT)

Exercise pattern: 5-minute warm-up, followed by 20 min of low-intensity continuous training, and a 5-minute cool-down. During the 20-minute main training phase, patients were required to maintain their heart rate stably below 55% HR_peak_ throughout. The therapist continuously and dynamically adjusted the equipment speed to keep the patient's heart rate within this target range, ensuring the low-intensity and continuous nature of the exercise. The total exercise duration, frequency, number of sessions, and equipment were identical between the two groups, only the target heart rate intensity range differed.

### Outcome measures

Participants were assessed at two time points: (1) at baseline, approximately 1 day before initiating the intervention, and (2) at post-treatment, within 1 week after the 4-week intervention period. The assessment included evaluating the cardiopulmonary function, ability of daily living (ADL), and lower extremity motor function. The primary outcome measure was VO_2peak_ (mL·kg^−1^·min^−1^) measured using a graded, symptom-limited maximal cardiopulmonary exercise test (CPET) in an incremental cycle ergometer (MasterScreen; Ergoline, Germany). The metabolic cart was calibrated before testing. The AHA guidelines state that in order to VO_2peak_, one must meet any of the following criteria: VO_2_ reaching a steady state, HR nearing the estimated maximum age-related heart rate, or peak respiratory exchange ratio (RER_peak_) reaching 1.15 or higher. In addition, HR_peak_, the highest registered heart rate during the VO_2peak_ test, was recorded as the intensity criterion. The criteria for exercise termination of the CPET were as follows: (1) The participant still can't maintain the cadence required by the experiment under encouragement; (2) respiratory exchange rate > 1.1; (3)Borg score > 17, and the participant reported that it was “very hard”; (4) The participant has chest tightness, unbearable dyspnea and abnormal ECG.

The secondary outcomes included oxygen uptake at anaerobic threshold (VO_2AT_), cardiac function parameters, ADL indices, and lower-extremity motor function. Anaerobic threshold (AT) point was identified using a combination of the V-slope analysis and ventilatory equivalents methods. Two independent raters determined the AT, and discrepancies were resolved by consensus.

The Barthel Index comprises 10 items. Each item is scored based on the level of assistance required and the time spent performing the activity, yielding a score of 15, 10, 5, or 0 points. The total score ranges from 0 to 100. A BI score of 60 was used as the cutoff to distinguish basic functional independence (BI ≥ 60) from moderate-to-severe functional dependence (B＜60) in activities of daily living (26). Baseline anthropometric measurements, including body weight and height, were obtained using calibrated instruments. BMI was subsequently derived using the standard formula: weight (kg)/[height (m)]^2^. Consistent with the guidelines issued by the Working Group on Obesity in China, participants were categorized based on BMI cut-offs of 24 kg/m^2^ for overweight and 28 kg/m^2^ for obesity (27). Lower limb motor function was assessed using the Fugl-Meyer Assessment (FMA-LL), a 17-item scale evaluating reflex activity, synergy patterns, and voluntary movement coordination, with total scores ranging from 0 to 34, where higher scores indicate better motor performance (28). All tests were performed by standardized trained physicians or exercise therapists in the cardiopulmonary rehabilitation unit blinded to the group assignments and previous test results.

### Statistical analysis

Continuous variables with a normal distribution were expressed as mean ± standard deviation (SD). Categorical variables in terms of demographic and clinical characteristics were expressed as frequencies or percentages. A new variable called “change score” was created, which is the difference between the post and pre-treatment values. The changes in score data with non-normal distribution are expressed as median (interquartile range). Missing data in this study were minimal, with all key variables having less than 5% missingness. We used multiple imputation to handle these missing values in order to minimize potential bias and maintain statistical power. The missing data mechanism was assumed to be missing at random (MAR). The imputation model included all variables used in the analysis as well as covariates potentially associated with missingness. Continuous variables were imputed using the predictive mean matching (pmm) method. Data analyses were performed using SPSS (version 27.0) and R (version 4.5.2). Key R packages used included ‘mice’ for multiple imputation and ‘dplyr’ for data management. The Independent-sample t-test or Mann–Whitney U test was used to examine whether the outcome measures of HIIT differed significantly from those of LICT. The paired-sample t-test or Wilcoxon signed-rank test was used to evaluate the difference in outcome measures between baseline and post-intervention. Differences were considered statistically significant when the P-value was less than 0.05.

## Results

A total of 48 patients were included in this study. Eight patients were lost to follow-up due to unplanned discharge (*n* = 3) or voluntary withdrawal (*n* = 5). Consequently, 40 patients were ultimately included in the final analysis. Table 1 shows the baseline characteristics of the groups. Of 40 recruited participants, 33 (82.5%) were males. 20 were allocated to the intervention group (18 males) and 20 to the control group (15 males). The mean age was 51 and 56 years in the intervention and control groups, respectively. No differences were found between the two groups regarding clinical messages (*P* > 0.05), and no adverse events related to the intervention were recorded during the study. Baseline comparisons revealed no significant intergroup differences in all functional levels, which shows that baseline characteristics were well matched between the HIIT and LICT groups.

**Table 1:** Characteristics of the LICT and HIIT Group At Baseline .

Variables	LICT (*n* = 20) Control	HIIT (*n* = 20) Intervention	P value
Gender: male, n (%)	15（75%）	18（90%）	0.407
Age in years at inclusion	55.79 ± 8.88	50.94 ± 7.86	0.075
BMI（kg/m^2^）	24.56 ± 2.38	25.10 ± 3.58	0.574
Clinical messages			
Stroke pathology (Ischemic), n (%)	16（80%）	11（55%）	0.176
Stroke duration(days)	17.5（14.75，59.00）	52.58（17.25，98.00）	0.151
Hypertension, n (%)	15（75%）	16（80%）	1.000
Diabetes, n (%)	8（40%）	6（30%）	0.741
Functional level			
Brunnstrom hand	2.00（1.00，3.00）	1.00 (1.00，3.00)	0.545
Brunnstrom upper extremity	2.00（2.00，3.00）	2.50（1.00，3.00）	0.726
Brunnstrom lower extremity	3.00（2.75，5.00）	3.00（3.00，4.00）	0.977
mRS	4.00（4.00，4.00）	4.00（3.00，4.00）	0.192
FMA-LL	16.80 ± 9.20	14.10 ± 8.39	0.338
BI	50.75 ± 18.94	56.50 ± 23.90	0.404
NIHSS	7.25 ± 4.19	6.00 ± 2.47	0.258
Cardiac function			
VO_2AT_(mL·kg^−1^·min^−1^)	9.26 ± 1.90	9.61 ± 1.75	0.548
VO_2peak_(mL·kg^−1^·min^−1^)	12.23 ± 2.06	13.59 ± 2.23	0.054

mRS: modified rankin scale; FMA-LL: Fugl-Meyer assessment lower limb; BI: Barthel Index; NIHSS: National Institute of Health stroke scale; VO_2AT_: oxygen uptake at anaerobic threshold; VO_2peak_: Peak oxygen uptake.

Values are shown as median (interquartile range), or Mean ± SD.

### Change in CRF in response to exercise training

The comparison of the primary and secondary outcome measures between the LICT and HIIT groups is presented in Table 2. Change scores were also calculated to represent the degree of improvement post-intervention compared to baseline.

**Table 2:** Comparison of primary and secondary outcome measures between LICT group and HIIT group.

Outcome measures	LICT (*n* = 20)				HIIT (*n* = 20)					
	Baseline	Post-intervention	Change score	P_1_	Baseline	Post- intervention	Change score	P_2_	P_3_	P_4_
			Mean change（CI）				Mean change（CI）			Mean change（CI）
**Primary outcome**										
VO_2peak_(mL·kg^−1^·min^−1^)	12.23 ± 2.06	13.03 ± 2.07	0.80（−0.14, 1.74）	0.090	13.59 ± 2.23	14.73 ± 3.69	1.14 (−0.22, 2.51)	0.096	0.083	0.665 0.35 (−1.26,1.96)
Secondary outcomes										
VO_2AT_ (mL·kg^−1^·min^−1^)	9.26 ± 1.90	9.90 ± 2.25	0.64（−0.46, 1.73）	0.237	9.61 ± 1.75	9.72 ± 2.62	0.12 (−0.97, 1.20)	0.827	0.825	0.483 −0.52(−2.01,0.97)
mRS	3.00（3.00，3.00）	3.50（2.75，4.00）	0.50（−0.00, 1.00)	0.239	3.00（2.00，3.00）	3.50（3.00, 4.00）	1.00（1.00, 1.50）	0.002	0.681	0.050* 0.00(−0.00,1.00)
FMA-LL	16.80 ± 9.20	22.30 ± 8.17	5.50（2.20, 8.80）	0.002*	14.10 ± 8.39	17.85 ± 6.87	3.75（0.91, 6.59）	0.012*	0.070	0.406 −1.75(−5.97,2.47)
BI	50.75 ± 18.94	67.50 ± 19.77	16.75（9.74，23.76）	0.000*	56.50 ± 23.90	72.00 ± 17.87	15.50 (6.78, 24.22)	0.001*	0.455	0.816 −1.25(−12.09,9.59)
CPET index										
RER_peak_	1.02（0.98，1.11）	1.07（0.99，1.13）	−0.01(−0.02, 0.08)	0.322	1.08（1.04, 1.16）	1.15（1.10, 1.20）	0.05（−0.03, 0.09）	0.420	0.086	0.968–0.00(−0.07,0.07)
exercise time(min)	7.83（6.08，10.13）	8.43（6.30，9.59）	0.40(−1.04, 1.79)	0.571	10.18 (7.70, 10.66）	9.92（8.06, 10.46）	−0.45（−1.45, 1.68）	0.286	0.064	0.337 −0.75(−2.73,1.68)

VO_2peak_: Peak oxygen uptake; VO_2AT_: oxygen uptake at anaerobic threshold; mRS: modified Rankin Scale; FMA-LL: Fugl-Meyer assessment lower limb; BI: Barthel Index; RERpeak: Peak respiratory exchange ratio; Change score: Difference post-pre of outcome. P1: Comparison before and after interventions in LICT groups; P2: Comparison before and after interventions in HIIT groups; P3: Comparison between LICT groups and HIIT groups; P4: Comparison between LICT groups and HIIT groups (Change score). *:Statistical significance *p* < 0.05.

variables are presented as mean ± SD or median (95% CI).

Peak respiratory exchange ratio (RER_peak_) was approximately 1.1 for both groups at baseline (1.02 vs. 1.08) and after the intervention (1.07 vs. 1.15), indicating that participants exerted sufficient effort during the cardiopulmonary exercise test (CPET). The average exercise duration for each participant ranged from 7 to 10 min.

Within-group comparisons (baseline vs. after the 4-week intervention) showed that the improvement in VO_2peak_ did not reach statistical significance in either the LICT group (12.23 ± 2.06 vs. 13.03 ± 2.07mL⋅kg^−1^⋅min^−1^, *P* = 0.090) or the HIIT group (13.59 ± 2.23 vs. 14.73 ± 3.69mL⋅kg^−1^⋅min^−1^, *P* = 0.096). Furthermore, no significant difference was observed in the change in VO_2peak_ between the HIIT and LICT groups (*P* = 0.665).

For secondary outcome measures, BI and FMA-LL significantly improved after 4 weeks of intervention in both groups (*P* < 0.05), while improvement in other secondary outcome measures (VO_2AT_) was not significant.

### Subgroup analysis

Analysis of covariance (ANCOVA) revealed a statistically significant interaction effect between “group” and “BI stratification” (F = 5.306, *P* = 0.027, partial *η*^2^ = 0.128), indicating that the magnitude of improvement in VO_2peak_ varied depending on the intervention type and the patient's BI level. Therefore, a *post hoc* stratified analysis was performed based on the clinical cutoff values of the BI (<60 and ≥60) to evaluate differential effects of the exercise intervention across patients with different baseline activity levels.

As shown in Table 3. In patients with a BI ≥ 60, a statistically significant intergroup difference in post-treatment VO_2peak_ was observed between the HIIT and LICT groups (16.35 ± 3.53 vs. 12.74 ± 2.60 mL·kg⁻^1^·min⁻^1^, *P* = 0.020), whereas no such difference was found in patients with a BI < 60. Furthermore, within the HIIT group, patients with a BI ≥ 60 exhibited significantly higher post-intervention VO_₂peak_ than those with a BI < 60 (16.35 ± 3.53 vs. 12.76 ± 2.98 mL·kg⁻^1^·min⁻^1^, *P* = 0.024).

**Table 3:** Comparison of outcome measures At post-intervention between LICT group and HIIT group (according to BI levels on admission).

Outcome measures	Admission BI <60 (*n* = 21)		P_1_	Admission BI ≥60 (*n* = 19)		P_2_	P_3_
	LICT (*n* = 12)	HIIT (*n* = 9)		LICT (*n* = 8)	HIIT (*n* = 11)		
	Post-score	Post-score		Post-score	Post-score		
	Mean ± SD	Mean ± SD		Mean ± SD	Mean ± SD		
Primary outcome							
VO_2peak_(mL·kg^−1^·min^−1^)	13.23 ± 1.73	12.76 ± 2.98	0.677	12.74 ± 2.60	16.35 ± 3.53	0.020*	0.024*
Secondary outcomes							
VO_2AT_(mL·kg^−1^·min^−1^)	10.67 ± 1.59	8.31 ± 2.06	0.012*	8.73 ± 2.68	10.88 ± 2.52	0.097	0.022*
mRS^a^	4.00（3.75，4.00）	4.00（3.00，4.00）	0.822	2.50（1.00，3.00）	3.00 (2.00, 4.00)	0.121	0.113
FMA-LL	19.83 ± 6.55	17.33 ± 6.86	0.411	26.00 ± 9.37	18.27 ± 7.18	0.073	0.769
BI	57.50 ± 16.17	60.56 ± 13.79	0.646	82.50 ± 14.88	81.36 ± 15.51	0.874	0.005*

VO_2peak_: Peak oxygen uptake;VO_2AT_: oxygen uptake at anaerobic threshold; mRS: modified Rankin Scale; FMA-LL: Fugl-Meyer assessment lower limb; BI: Barthel Index.

P1: Comparison between the LICT and HIIT groups within the low ADL subgroup (BI < 60）; P2: Comparison between the LICT and HIIT groups within the high ADL subgroup (BI ≥ 60); P3: Comparison between low ADL (BI < 60) and high ADL (BI ≥ 60) subgroups within the HIIT group.

* Statistical significance *p* < 0.05.

a Data are presented as median (interquartile range).

Considering that BMI is also an important determinant of VO_2peak_, we further conducted a stratified analysis based on the Overweight threshold (BMI ＞24 kg/m^2^). Among patients with a BMI ≤24 kg/m^2^, a statistically significant intergroup difference in post-treatment VO_2peak_ was observed between the HIIT and LICT groups (16.90 ± 3.11 vs. 13.51 ± 2.02 mL·kg^−1^·min^−1^, *P* = 0.016)，and within the HIIT group, patients with a BMI ≤24 kg/m^2^ exhibited significantly higher post-intervention VO_₂peak_ than those with a BMI >24 kg/m^2^ (16.90 ± 3.11 vs. 12.95 ± 3.24 mL⋅kg−1⋅min−1, *P* = 0.013). (As shown in Table 4)

**Table 4:** Comparison of outcome measures At post-intervention between LICT group and HIIT group (according to BMI levels on admission).

Outcome measures	Admission BMI≤24 (*n* = 18)		P_1_	Admission BMI＞24(*n* = 22)		P_2_	P_3_
	LICT (*n* = 9)	HIIT (*n* = 9)		LICT (*n* = 11)	HIIT (*n* = 11)		
	Post-score	Post-score		Post-score	Post-score		
	Mean ± SD	Mean ± SD		Mean ± SD	Mean ± SD		
Primary outcome							
VO_2peak_(mL·kg^−1^·min^−1^)	13.51 ± 2.02	16.90 ± 3.11	0.016*	12.65 ± 2.12	12.95 ± 3.24	0.795	0.013*
Secondary outcomes							
VO_2AT_(mL·kg^−1^·min^−1^)	10.45 ± 2.97	11.13 ± 2.64	0.615	9.44 ± 1.44	8.57 ± 2.05	0.265	0.031*
mRS^a^	4.00 (3.00,4.00)	3.00 (3.00, 4.00)	0.702	3.00 (2.50, 4.00)	4.00（3.00, 4.00)	0.366	0.381
FMA-LL	19.44 ± 7.80	19.44 ± 7.75	1.000	24.64 ± 8.05	16.55 ± 6.12	0.016*	0.375
BI	61.11 ± 21.18	79.44 ± 14.02	0.048*	72.73 ± 17.80	65.91 ± 18.95	0.395	0.083

BMI was categorized into normal-weight (≤24 kg/m^2^) and overweight/obese (>24 kg/m^2^) P1: Comparison between the LICT and HIIT groups within the normal-weight subgroup (BMI ≤24); P2: Comparison between the LICT and HIIT groups within the overweight/obese subgroup (BMI ≥24); P3: Comparison between normal-weight and overweight/obese subgroups in the HIIT group.

*:Statistical significance *p* < 0.05.

a Data are presented as median (interquartile range).

## Discussion

This is a randomized controlled trial (RCT) to evaluate the effects of HIIT on cardiorespiratory fitness in non-ambulatory stroke patients. Our results indicate that a 4-week short-interval HIIT program improves VO_2peak_, but does not demonstrate a significant advantage over LICT. However, stratified analyses suggest that the superior effects of HIIT may be closely associated with baseline activities and BMI levels.

In this study, the HIIT group achieved a post-intervention VO_2peak_ of 14.73 ± 3.69 mL·kg^−1^·min^−1^, compared to 13.03 ± 2.07 mL·kg^−1^·min^−1^ in the LICT group. Although the mean change in the HIIT group exceeded the minimal clinically important difference (MCID), neither within-group nor between-group differences reached statistical significance.​This aligns with Balady et al, who reported that an 8-week HIIT program combined with standard care was not superior to standard care alone in improving VO_2peak_ (*P* = 0.665) (29, 30). The lack of significant improvement may be attributed to the duration of the intervals，more pronounced adaptations in VO_2peak_ might require longer high-intensity bouts (31, 32). For instance, Gjellesvik et al. employed a 4 × 4 min protocol (at 85%–95% HRmax) with 3 min active recovery and observed significant gains in VO_2max_ (22). In contrast, considering that non-ambulatory patients may not tolerate prolonged high-intensity exercise, our study utilized 1 × 1 min intervals at 85%–95% HR_peak_ (20). This design parallels Boyne et al.'s approach of 30-s sprints interspersed with 60-s recovery, where no significant VO_2peak_ changes were detected (23). These findings suggest that a sufficient duration of high-intensity stimulus per bout may be a prerequisite for inducing robust cardiovascular adaptation. Additionally, according to ACSM guidelines, initial cardiovascular adaptations typically emerge 2 to 6 weeks after training onset; thus, a training period shorter than 6 weeks may have been insufficient to elicit significant physiological remodeling in our previously untrained cohort (33, 34).

The heterogeneity in the effects of exercise on cardiovascular biomarkers may offer an alternative explanation for the lack of significant between-group differences between HIIT and LICT observed in this study. Some studies have suggested that the improvement in cardiorespiratory fitness (CRF) induced by HIIT may be attributable not only to skeletal muscle metabolic adaptation and mitochondrial biogenesis, but also to the modulation of vascular endothelial function and inflammatory status (35, 36). However, existing evidence has not consistently supported a more pronounced superiority of HIIT over continuous aerobic training in these two aspects (37). With regard to vascular function, previous studies in stroke patients have shown that HIIT did not produce significant improvements in either carotid-femoral pulse wave velocity (cfPWV) or glyceryl trinitrate-mediated dilation (GTN) (35, 38). Although some studies have suggested that HIIT is superior to MICT in improving flow-mediated dilation (FMD), these studies are characterized by considerable heterogeneity and have not been conducted in stroke populations, leaving the generalizability of their findings to stroke patients uncertain. With regard to inflammatory markers, in patients with type 2 diabetes mellitus (T2DM), improvements in inflammatory indicators such as IL-6 and NF-*κ*B also appear to be more closely associated with aerobic training (AT) than with HIIT (39). Therefore, future studies are warranted to examine the specific effects of HIIT on vascular endothelial function and inflammatory markers in stroke populations, and to explore the potential mediating pathways between these markers and improvements in CRF, which may help explain the lack of distinct between-group outcomes for HIIT and LICT observed in the present study.

Subgroup analysis based on the Barthel Index (BI) provided critical insights into the heterogeneity of treatment effects. In the HIIT group, participants with BI ≥60 showed significantly greater improvements in VO_2peak_ compared to those with BI <60. Furthermore, the HIIT group outperformed the LICT group exclusively within the BI ≥60 group. This disparity likely stems from fundamental differences in physiological capacity and neurorestorative potential (12). A BI ≥60 indicates not only basic self-care ability but also implies adequate muscle strength, range of motion, and coordination. Such a physiological foundation likely enabled the cardiorespiratory system to better tolerate the stress of high-intensity target heart rate zones (21). Moreover, higher functional status allows patients to execute and consolidate correct movement patterns during repetitive training, creating a positive feedback loop that enhances the neuroplastic benefits induced by aerobic exercise (40). Conversely, patients with BI <60, constrained by limited functional capacity, often experienced premature fatigue, compromising training quality and preventing them from reaching the requisite stimulus threshold (22, 41). Supporting this, a study on ICU-acquired weakness found that HIIT failed to significantly improve the 6-minute walk distance (159.5 ± 64.9 m vs. 120.4 ± 60.4 m; *P* = .057) or the modified Barthel Index (*Δ*28.9 ± 16.0vs. *Δ*25.0 ± 10.5; *P* = .341) compared to MICT in a functionally limited population. In summary, baseline BI level appears to be a key moderator predicting HIIT efficacy (20). Future rigorously designed RCTs are warranted to validate BI as a stratification tool and to explore optimized interventions for low-BI populations.

Obesity, one of the traditional risk factors for cardiovascular diseases, is prevalent among populations with stroke and other cardiovascular disorders, and can exacerbate oxidative stress, insulin resistance, and vascular endothelial dysfunction (42). These pathophysiological mechanisms are closely associated with the process of aerobic exercise-induced cardiovascular adaptation, suggesting that obesity may act as an effect modifier that influences the therapeutic efficacy of exercise interventions (39).Accordingly, the present study further conducted subgroup analyses stratified by BMI. The results demonstrated that the magnitude of improvement in CRF following HIIT intervention was relatively more pronounced in stroke patients who did not meet the overweight criterion (BMI <24 kg/m^2^). This phenomenon may be attributable to the greater cardiac output (CO) and enhanced peripheral oxygen utilization elicited by HIIT: the sustained cardiovascular load imposed by high-intensity exercise may promote adaptive remodeling of cardiac structure and function, thereby further enhancing CRF (36, 43). In contrast, the degree of improvement was relatively limited in the overweight/obese subgroup, which may be associated with visceral adipose accumulation related to elevated BMI. Such accumulation not only induces chronic low-grade inflammation but is also considered to be linked to impaired motor function and unfavorable overall rehabilitation outcomes (44). It should be noted that, constrained by the sample size of the subgroups and the exploratory nature of this finding, the above conclusions remain to be further validated in prospective studies with larger sample sizes.

Based on the findings of the present study, individualized exercise prescriptions should be developed in clinical practice for stroke patients with impaired walking ability according to their baseline functional status and body weight. For patients with poorer baseline functional capacity or obesity, LICT may still effectively improve cardiopulmonary fitness and motor function, whereas HIIT may provide greater improvements in cardiorespiratory fitness for patients with relatively preserved functional capacity or normal body weight. In addition, the specific HIIT protocol requires further optimization. The short-interval protocol used in this study (1 min high-intensity alternating with 1 min low-intensity exercise) may not provide sufficient recovery time for patients who are prone to fatigue (BI <60 or BMI ≥24), thereby limiting training efficacy. For these patients, a long-interval HIIT protocol (e.g., 30 s high-intensity exercise followed by 2–3 min low-intensity recovery) may be more appropriate (45, 46). This issue warrants further investigation in future studies.

This study has several limitations that should be acknowledged. First, the relatively small sample size may have limited the statistical power to detect smaller but clinically meaningful differences between HIIT and LICT. Second, constrained by the typical duration of hospitalization, the intervention period was only 4 weeks, which may be insufficient to observe the long-term effects and sustainability of the training benefits on cardiorespiratory fitness and functional recovery. Third, given the nature of exercise interventions, participants were inevitably aware of their group allocation, and the physical therapists delivering the interventions were not blinded to treatment assignment. This open-label design introduces a risk of performance bias and detection bias, which should be considered when interpreting the results. Fourth, the subgroup analyses by BMI and BI were not pre-specified in the trial registration, and no correction for multiple comparisons was applied. These findings should be interpreted as exploratory. These limitations underscore the necessity for future larger-scale, multicenter randomized controlled trials with extended intervention and follow-up periods, incorporating blinded outcome assessment, to confirm and broaden the applicability of the results.

## Conclusion

In conclusion, among non-ambulatory stroke patients, the 4-week HIIT program did not show a statistically significant difference from LICT in overall improvement of cardiorespiratory fitness. However, exploratory subgroup analyses suggested that patients with higher baseline activity levels or non-obese status may benefit more from HIIT. This finding suggests that individualized exercise prescription may hold potential value in clinical practice, whereby training modality and intensity might need to be adjusted according to patients’ baseline functional status and body composition. However, its precise efficacy warrants further confirmation in larger-scale trials.

## Data Availability

The original contributions presented in the study are included in the article/Supplementary Material, further inquiries can be directed to the corresponding authors.
